# Lupus Cystitis, From Myth to Reality: A Narrative Review

**DOI:** 10.7759/cureus.20409

**Published:** 2021-12-14

**Authors:** Juan Camilo Santacruz, Sandra Pulido, Angelo Arzuaga, Marta Juliana Mantilla, John Londono

**Affiliations:** 1 Spondyloarthropathies Research Group, Universidad de la Sabana, Chía, COL; 2 Rheumatology Department, Universidad Militar Nueva Granada, Bogotá, COL

**Keywords:** methylprednisolone, immune complexes, cyclophosphamide, lupus cystitis, systemic lupus erythematosus disease

## Abstract

Systemic lupus erythematosus is a multisystemic disease that usually involves the urinary tract, often in the form of lupus nephritis. However, another form of compromise of this system is lupus cystitis, which, despite being an unusual condition, turns out to be a challenging diagnosis due to the spectrum of nonspecific abdominal and urinary symptoms. Although the exact pathophysiological mechanism of bladder inflammation remains to be established, the role of small vessel vasculitis measured by immune complexes continues to be supported as a central axis for considering possible therapeutic targets. Additionally, there are no clinical studies that dictate a guideline regarding its treatment, however, the evidence from most cases described in the literature suggests the initiation of pulses of methylprednisolone and cyclophosphamide in treatment regimens similar to those of lupus nephritis. Despite its low prevalence, obstructive complications and kidney damage can lead to increased morbidity and mortality.

## Introduction and background

Systemic lupus erythematosus (SLE) is a systemic autoimmune disease of variable severity with a tendency to flare up over the course of its evolution. Immunological alterations, particularly the production of various antinuclear antibodies (ANA), are a determining feature of the disease. Both the innate and adaptive immune systems are involved in its pathophysiology, as well as the interaction between genes with environmental factors that cause sustained immunological alterations against autologous nucleic acids [1]. The tissue damage attributed to the disease is caused by autoantibodies or the deposition of immune complexes that are located mainly in the kidneys, heart, blood vessels, central nervous system, skin, lungs, muscles, joints, and bladder [2]. Lupus cystitis is a rare but significant complication within SLE, which, on some occasions, can cause permanent bladder dysfunction, leading to irreversible deterioration of kidney function [3]. It is characterized by a spectrum of abdominal and urinary symptoms that are not specific for its diagnosis so that, in most cases, its clinical suspicion can be ignored with the consequent progression of obstructive uropathy attributed to the progressive decrease of the bladder capacity due to fibrosis and inflammation. Without treatment, lupus cystitis is associated with complications such as intestinal pseudo-obstruction, hydroureteronephrosis, ureteritis, and mesenteric vasculitis. It is estimated that lupus cystitis can affect between 0.01% to 2% of all patients with SLE, of which 92% are women [4]. Most of the reported cases have been described in East Asia, especially Japan where it appears to be slightly more prevalent [5].

## Review

Methods

**Figure 1:**
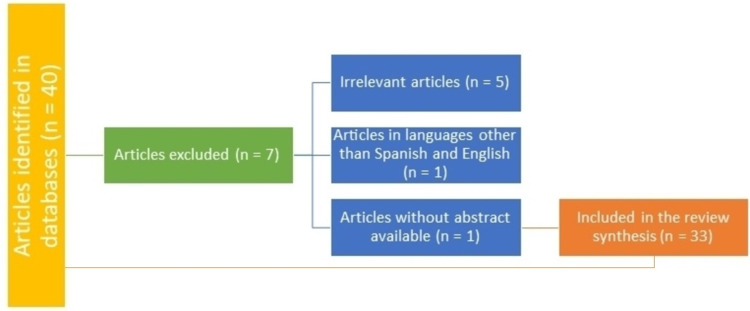
Search flow diagram

A non-systematic narrative review of the literature developed in English and Spanish was carried out, following the objective of having the most representative information for the referenced articles until 2021 in primary databases such as Pubmed, academic Google, LILACS, ScienceDirect, and EMBASE. The MESH (medical subject headings) terms used were: "lupus cystitis", "treatment", "refractory lupus cystitis", and "lupus interstitial cystitis" combining Boolean operators (AND, OR). Articles were included that were reports and/or series of cases of adult patients, reviews of topics, and narrative reviews that delved into the etiology of lupus cystitis, the immunological mechanisms involved in its pathophysiology, and the therapeutic effects achieved with the different immunosuppressive drugs. Below is a flow chart detailing the search strategy (Figure 1).

Pathophysiology

**Figure 2:**
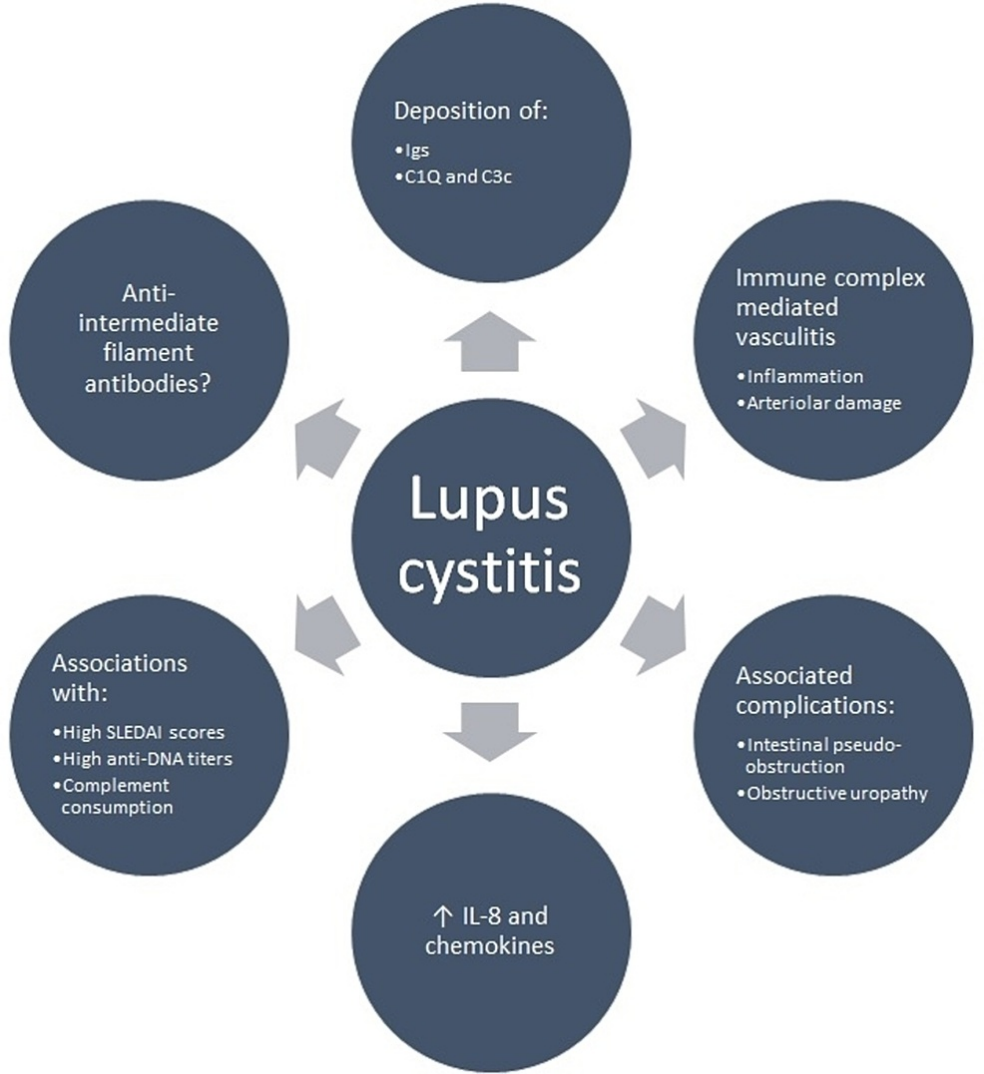
Central aspects of the pathophysiology of lupus cystitis Igs: immunoglobulins; SLEDAI: Systemic Lupus Erythematosus Disease Activity Index

The pathophysiological mechanisms involved in lupus cystitis are not clearly understood. It has been suggested that immune complex-mediated vasculitis may play an important role due to the deposition of immunoglobulin G (IgG), IgM, IgA, C1Q, and C3c in the arterioles located in the bladder of some patients [6-7]. Higher concentrations of certain interleukins, such as interleukin 8 (IL-8) and monocyte-activating chemotactic factor (MCAF) have also been described, also showing a lower concentration of these cytokines after treatment [8]. Although there does not appear to be an adequate correlation between autoantibody production and the genesis of lupus cystitis, one study demonstrated that anti-intermediate filament antibodies were disease-specific [9]. Bladder smooth muscle dyskinesia is caused by direct immunological mechanisms, further supporting the participation of immune complexes in the origin of this entity. These findings have provided important information to achieve a better understanding of its pathophysiological mechanism and thus obtain better therapeutic responses (Figure 2). Table 1 describes the most important risk factors associated with its presentation.

**Table 1:** Risk factors associated with the presentation of lupus cystitis ESR: erythrocyte sedimentation rate; SLE: systemic lupus erythematosus; SLEDAI: Systemic Lupus Erythematosus Disease Activity Index

Risk factor´s	Reference
Female sex	[10]
Anti-dsDNA antibodies present	[10]
Concomitant neuropsychiatric SLE	[8]
Concomitant lupus enteritis	[8]
History of mesenteric vasculitis	[7]
Vomiting with weight loss	[3]
High SLEDAI score (≥6 points)	[8]
Low C3 level	[6]
High levels of ESR at admission	[11]

Clinical symptoms and complications

Lupus cystitis can precede the diagnosis of SLE on some occasions [10]. During the disease, a progressive decrease in bladder capacity due to fibrosis is observed together with the thinning of its wall [11]. The first symptoms can be urinary or gastrointestinal, the latter being the most frequently observed given their coexistence with lupus enteritis. Patients may present with abdominal pain, watery diarrhea, constipation, and nausea, sometimes causing recurrent emesis and weight loss [12-13]. The predominant symptoms of the urinary tract are urgency and frequency, usually accompanied by pain or discomfort in the suprapubic region [14-15]. Frequency, dysuria, nocturia, and lower abdominal pain are the cardinal symptoms of cystitis, achieving some improvement during urination [16]. Urinalysis is usually normal in most cases, although the presence of hematuria is occasionally observed [17]. A serious consequence of lupus cystitis is hydroureteronephrosis, which occurs as a result of the reduction of the vesicoureteral outlet space caused by inflammatory edema and fibrosis [18]. Prolonged obstructive uropathy, without intervention to ensure the outflow of the pyelocalyceal system, can result in irreversible kidney damage [19]. Abdominal pelvic CT is the method of choice for the diagnosis and follow-up of complications (hydroureter, hydronephrosis, ureterectasia, etc). The most important findings that can be visualized are; bladder wall thickening, intestinal wall thickening, decreased bladder capacity, and ascites [20-21]. Cystoscopy may show diffuse inflammation of the bladder with erythema and bleeding at the trigone. Histopathology shows a chronic inflammatory infiltrate in the subepithelium, which is initially scant until it progressively increases, ultimately leading to fibrosis and urethral obstruction [22-23].

Treatment

**Figure 3:**
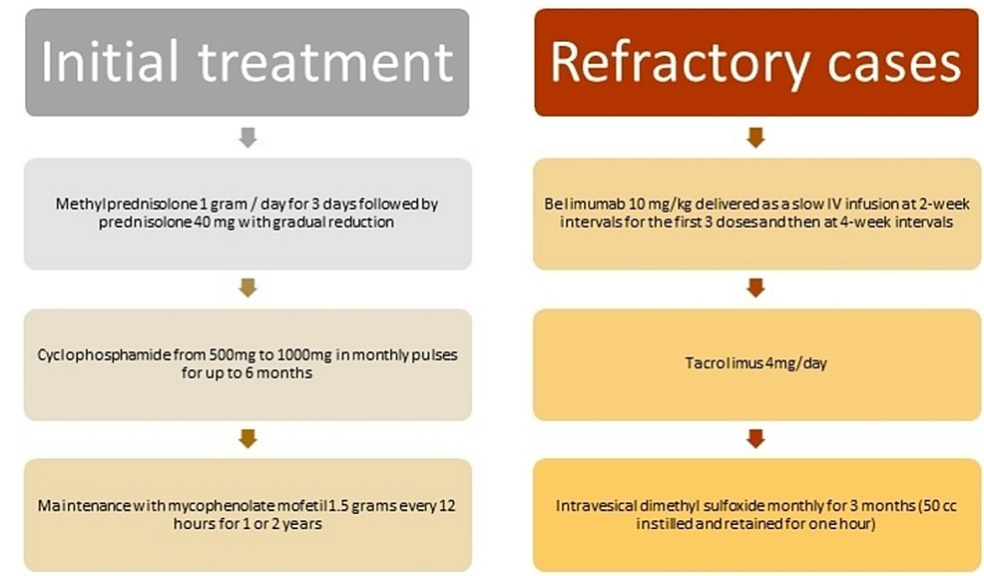
Treatment of lupus cystitis and refractory lupus cystitis

Currently, there is no guideline about a therapeutic guide for avoiding the complications of lupus cystitis. However, glucocorticoids and some immunosuppressive drugs, such as mycophenolate and cyclophosphamide, have been used with good results in most of the reported cases. The favorable effect of starting treatment with pulses of methylprednisolone is a consistent finding in the cases described, but with a great variation in the required doses [24-25]. Concerning the other immunosuppressants, the doses have also varied according to the severity of the presentation. The experience for the treatment of refractory cases is also limited, however, there are cases with favorable clinical outcomes with the addition of belimumab, tacrolimus, and intravesical dimethyl sulfoxide [26-28]. The diagnosis of lupus cystitis can be challenging, particularly in developing countries, where chronic infections, such as genitourinary tuberculosis, are more frequent [29]. The premise should be to exclude this condition, as well as other infectious causes, before making the diagnosis of lupus cystitis and starting immunosuppressive treatment. Early initiation of therapy is essential to avoid associated obstructive complications and thus reduce the possibility of requiring renal replacement therapy at some point during its presentation. A treatment algorithm based on the good results obtained from the most representative cases is described below (Figure 3), and Table 2 describes the doses, clinical characteristics, and complications that were evaluated in each one, including the references [30-33].

**Table 2:** Immunosuppressive drugs used in cases with favorable clinical outcomes CYC: cyclophosphamide; MMF: mycophenolate mofetil; IV: intravenous

Author and year	Drug and dosage	Initial symptoms	Complications	Reference
Castaño-González, et al., 2019	Methylprednisolone 1 gram daily for 3 days and CYC at 750 mg/m^2^ (IV)	Abdominal pain and dysuria	Intestinal pseudo-obstruction and right hydroureteronephrosis	[30]
Kaneshita S, et al., 2017	Methylprednisolone 60 mg daily	Vomiting and diarrhea	Hydronephrosis of the right kidney	[31]
Harris CR, et al., 2015	Methylprednisolone and MMF (doses not mentioned)	Abdominal pain, facial rash, dysuria, and fatigue	Mild bilateral hydroureteronephrosis	[22]
Mukhopadhyay S, et al., 2015	Methylprednisolone 1 gram daily for 3 days and CYC 500 mg every 2 weeks for 6 doses (IV)	Fever, abdominal pain, urgency, and vomiting	Bilateral hydronephrosis and ascites	[3]
Kinoshita K, et al., 2008	Methylprednisolone 750-1000 mg for 3 days (IV)	Nausea, vomiting, abdominal pain, and diarrhea	Bilateral hydronephrosis	[32]
Koh JH, et al., 2015	Prednisone with an average of 60 mg per day, vincristine, MMF, and CYC (without specified dose)	Nausea, vomiting, abdominal pain, and diarrhea	Ureteritis, lupus mesenteric vasculitis	[33]

## Conclusions

Even though lupus cystitis is an entity with very low prevalence, ignoring its diagnosis would lead to devastating complications derived from the obstruction of the urinary system, resulting in irreversible kidney damage. Among the risk factors to present it, the following stand out: female sex, the presence of anti-DNA, the consumption of C3, a high SLEDAI score, and the coexistence of enteritis or mesenteric vasculitis due to SLE. It is essential to evaluate gastrointestinal symptoms in the first instance since they are the ones that occur most frequently. Abdominopelvic CT continues to be the method of choice for establishing the diagnosis and evaluating associated complications. There is no treatment guide based on clinical studies, however, current evidence supports the use of pulses of methylprednisolone and cyclophosphamide, obtaining good results in most of the cases described. The good therapeutic response observed with these drugs is supported by their pathophysiological model whose the central axis is immunocomplex-mediated vasculitis, being very similar to what happens in lupus nephritis. Additionally, refractory cases have been treated with drugs similar to those used in refractory lupus nephritis such as belimumab and tacrolimus. It is vitally important to rule out infectious causes before starting immunosuppression treatment, particularly genitourinary tuberculosis. The reason for its higher prevalence in certain countries of the Asian continent is unknown, although it is presumed that some hereditary or ethnic-related conditions may be predisposing factors. Early diagnosis and treatment of this condition have a great impact on achieving favorable clinical outcomes.
